# Durable and deep response to CVD chemotherapy in SDHB-mutated metastatic paraganglioma: case report

**DOI:** 10.3389/fendo.2024.1483516

**Published:** 2024-12-18

**Authors:** Chenyan Zhang, Yuanfeng Wei, Ke Cheng, Dan Cao

**Affiliations:** Division of Abdominal Tumor, Department of Medical Oncology, Cancer Center and State Key Laboratory of Biological Therapy, West China Hospital, Sichuan University, Chengdu, Sichuan, China

**Keywords:** SDHB-mutation, metastatic paraganglioma, CVD chemotherapy, case report, hypoglycemia

## Abstract

**Introduction:**

Succinate dehydrogenase subunit B (SDHB)-mutated paragangliomas (PGLs) are rare neuroendocrine tumors characterized by increased malignancy, readily metastasizing, and poorer prognosis. Here we report a case of SDHB-mutated metastatic PGL, wherein the patient showed significant tumor shrinkage and complete symptom remission following chemotherapy. We aim to contribute additional evidence to the existing knowledge associated with SDHB-mutated PGLs.

**Case report:**

A 40-year-old male patient presented with recurrent hypoglycemia and hypertension crisis. Imaging revealed a huge left retroperitoneal tumor and multiple diffuse metastases in lungs. Catecholamine was also elevated, aligning with a diagnosis of metastatic PGL. Pathology also confirmed this diagnosis. Additionally, the immunohistochemistry indicated negative expression of SDHB and gene test showed somatic SDHB mutation. Given the SDHB mutation, cyclophosphamide-vincristine-dacarbazine (CVD) chemotherapy was initiated in critical conditions. Subsequently, a significant tumor shrinkage and complete biochemical response were observed after two treatment cycles. In September 2024, CT scan revealed new pulmonary lesions. The progression-free survival (PFS) with CVD chemotherapy was 24 months.

**Conclusion:**

This report reviews the distinct clinical and biochemical characteristics and treatment approaches of SDHB-mutated paragangliomas, emphasizing that the significance of incorporating both genetic testing and immunohistochemical analysis in clinical practice.

## Introduction

Paragangliomas (PGLs) are rare neuroendocrine tumors with high heritability (1). Around half of PGLs are linked to mutations in succinate dehydrogenase subunit x (SDHx) genes (2, 3). Among these, SDHB mutations are the most common (1). SDHB-mutated PGLs present distinct clinical and biochemical features that may guide personalized therapy (1, 4–6). Here, we report a case of SDHB-mutated metastatic PGL, demonstrating significant tumor shrinkage and complete symptom remission following cyclophosphamide-vincristine-dacarbazine (CVD) chemotherapy. This case aims to contribute further evidence to the understanding of SDHB-mutated PGLs.

## Case presentation

In August 2022, a 40-year-old male Asian patient, presented at the emergency department with syncope, diaphoresis, sialorrhea, absence of tic, and urinary and fecal incontinence. He displayed facial edema, a heart rate of 98 beats per minute (bpm), a blood pressure of 193/114 mm Hg, a respiratory rate of 20 breaths per minute, a body mass index of 24.2 kg/m^2^, and an Eastern Cooperative Oncology Group (ECOG) score of 2. 1 hours later, he gradually regained consciousness. Approximately 7 months prior to this event, he began experiencing recurring hypoglycemia at night. Over the preceding six months, he suffered recurrent headaches and his self-measured systolic blood pressure at onset exceeded 180 mmHg. Besides, there is no significant medical, familial, or psychosocial history.

**Figure 1:**
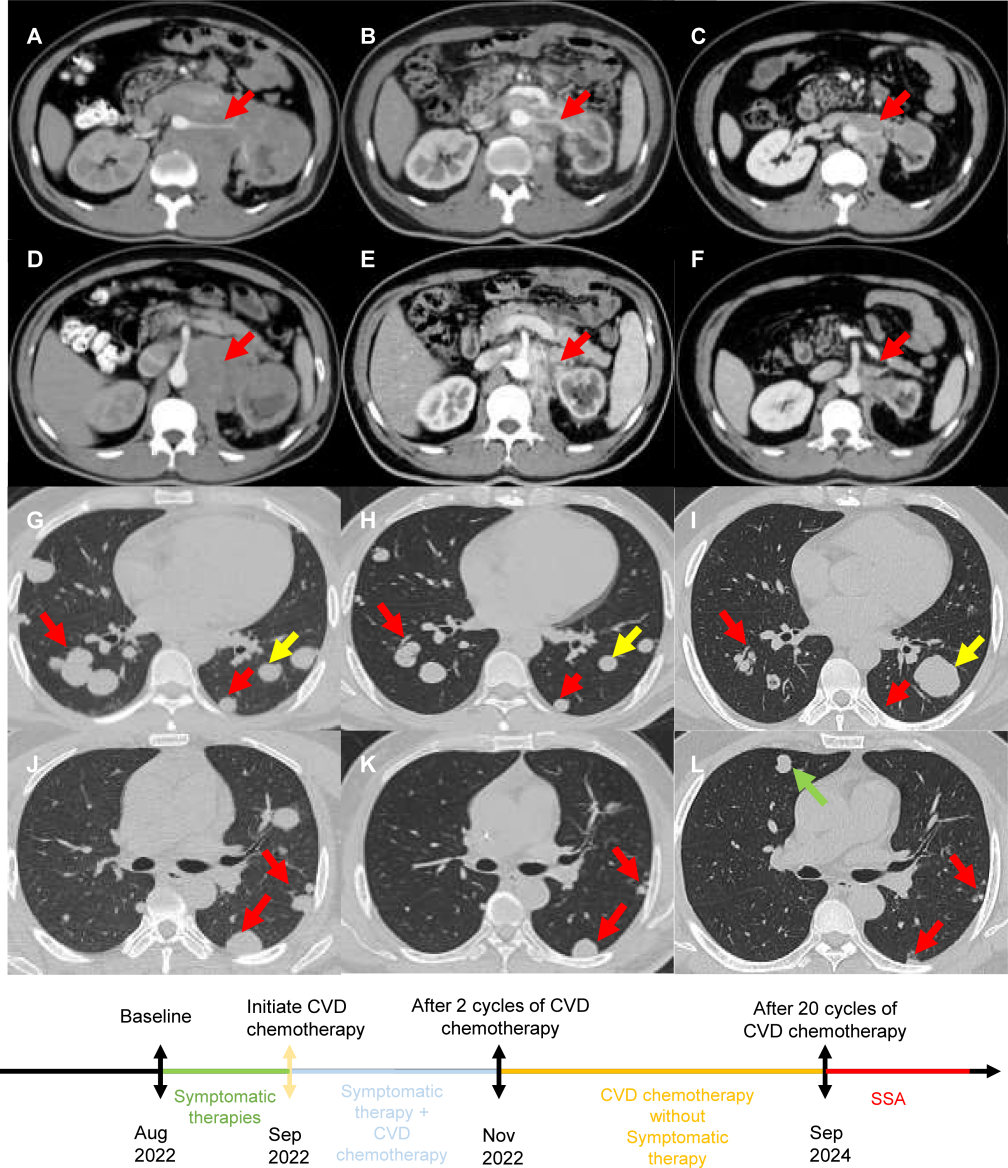
**(A, D, G, J)** Prior to CVD chemotherapy, CT revealed retroperitoneal lesions around the pancreas, liver, kidney and multiple diffuse lesions in both lungs. **(B, E, H, K)** After 2 cycles of CVD chemotherapy, CT revealed that retroperitoneal lesions around the pancreas, liver, kidney and multiple diffuse lesions in both lungs reduced in size. **(C, F, I, L)** After 20 cycles of CVD chemotherapy, CT revealed retroperitoneal lesions further reduced in size. Although a small amount of lung lesions enlarged and new lesions appeared, the majority of lung lesions reduced in number and size. [Red arrows indicate shrinking lesions, yellow arrows indicate enlarged lesions, and green arrows indicate new lesions.].

**Figure 2:**
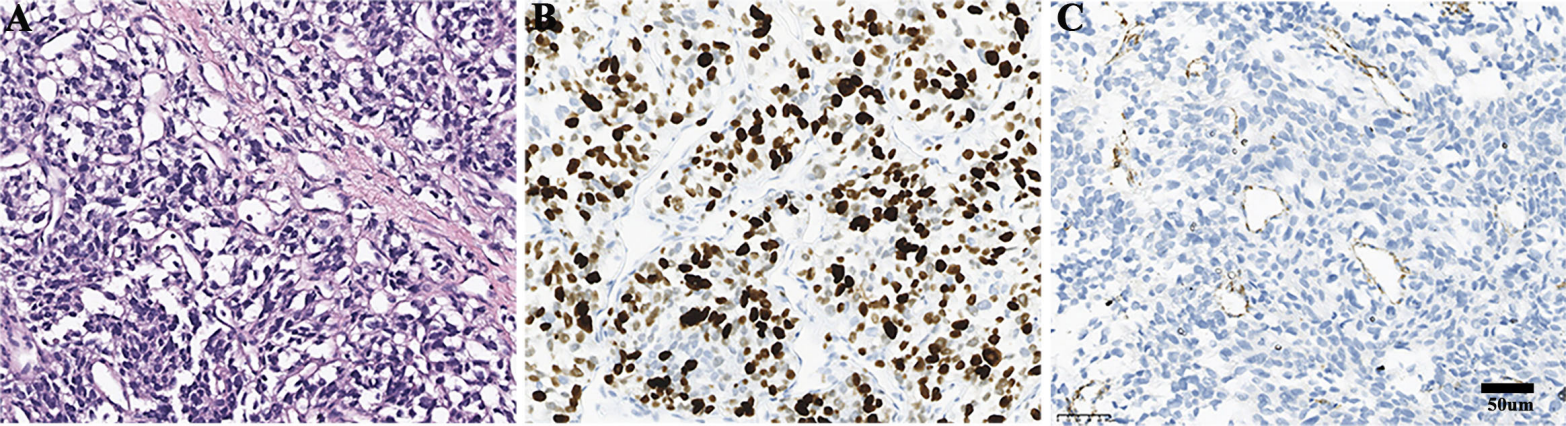
Pathological findings of the retroperitoneal lesion. **(A)** Haematoxylin and eosin staining. **(B)** Ki67 positive rate was 60%. **(C)** The expression of SDHB was negative.

**Figure 3:**
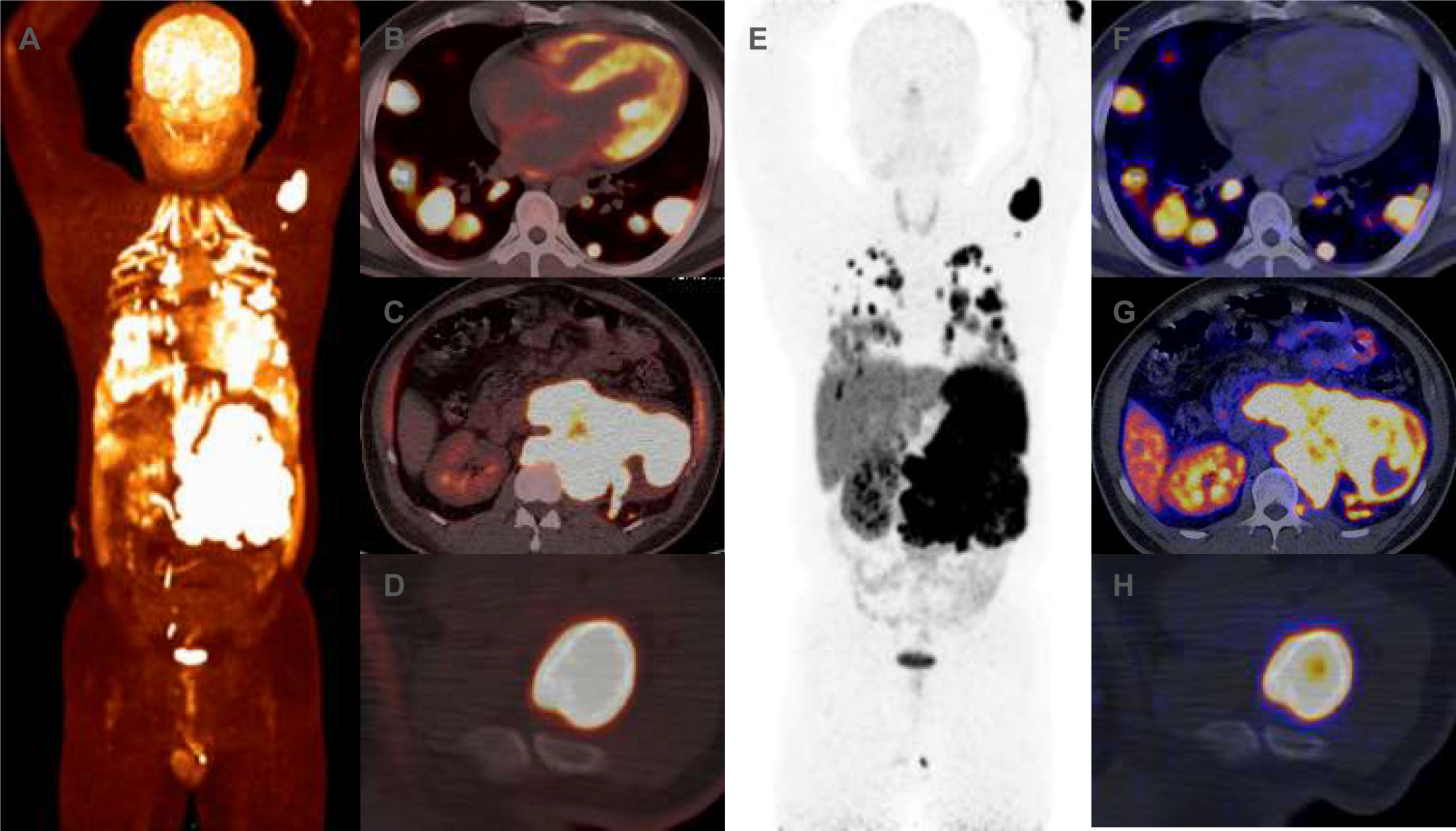
**(A–F)** Prior to CVD chemotherapy, 18F-FDG positron emission tomography (PET-CT) revealed increased uptake of 18F-FDG in the left retroperitoneal mass, lung masses and humeral head. The maximum cross-sectional area of the left retroperitoneal mass was approximately 166 × 99mm, with maximum SUV sizes of 21.49. **(G–L)** Prior to CVD chemotherapy, 68Ga-DOTATATE PET-CT revealed increase uptake of 68Ga-DOTATATE in the left retroperitoneal mass, lung masses and humeral head. The maximum SUV sizes of the left retroperitoneal mass was 36. 94.

Routine blood tests, liver and kidney function assessments, ECG, and cranial CT scans revealed no abnormalities. Serum levels of insulin, C-peptide, insulin-like growth factor I (IGF-I), and growth hormone (GH) were normal when blood glucose was 1.5 mmol/L (**Table 1**). However, thorax-abdomen CT identified a 14.1×9.7 cm left retroperitoneal mass and multiple pulmonary lesions (**Figures 1A, D, G, J**). Catecholamine, ACTH, and NSE levels were elevated (**Table 1**), aligning with a diagnosis of metastatic PGL. Although hyperglycemia is common in PPGL due to excessive catecholamine secretion, this patient experienced recurrent hypoglycemia, prompting us to further confirm the diagnosis through pathology. Additionally, imaging showed multiple lung metastases, ruling out curative surgery. Thus, the patient and family opted for a retroperitoneal lesion biopsy after discussing the biopsy risks. Fortunately, no adverse reactions occurred. Immunohistochemical analysis showed positive expression of Synaptophysin (Syn), Chromogranin A (CgA), and SSTR2, but negative for SDHB and S100 (**Figure 2**), with a Ki67 labeling index of 60%. Next-generation sequencing (NGS) revealed a somatic copy number loss of the SDHB gene. ^68^Ga-DOTATATE and ^18^F-FDG positron emission tomography (PET-CT) scans were conducted. The results revealed that the metastasis affected the lungs and skeletal sites, including the anterior segment of the left 7th rib, left scapula, and left humerus (**Figure 3**).

**Table 1:** Levels of serum insulin, C-peptide, IGF-I, GH and β-hydroxybutyrate during episode of hypoglycaemia.

Test	Value	Normal range	Blood glucose level at the time of measurement	Collection time
Insulin	<0.4	1.5uU/ml at least	1.5 mmol/L	during hypoglycaemia
C-peptide	0.031	0.3-1.3 nmol/L	1.5 mmol/L	during hypoglycaemia
IGF-I	39.53	107-216 ng/ml	1.5 mmol/L	during hypoglycaemia
Growth hormone (GH)	0.34	0.030-2.47 ng/ml	1.5 mmol/L	during hypoglycaemia
β-hydroxybutyrate	0.05	0.02 - 0.27 mmol/L	1.5 mmol/L	during hypoglycaemia
Norepinephrine	10.23	0-5.17 nmol/L	N/D	morning
Normetanephrine	12.86	0-0.71 nmol/L	N/D	morning
3-mexoxytyramine	25.83	0-18.4 pg/ml	N/D	morning
Epinephrine	0.26	0-0.34 nmol/L	N/D	morning
Dopamine	0.20	0-0.31 nmol/L	N/D	morning
ACTH	110.70	5-78 ng/L	N/D	morning
Cortisol	435.00	138-690 nmol/L	N/D	morning
NSE	70.30	0-20.4 ng/ml	N/D	morning

Despite 1 month of symptomatic therapies, including alpha blockade and intravenous fluid replacement, recurrent hypoglycemia and hypertensive crises persisted. The progression of the disease was presumed to be rapid based on the time when the patient became aware of symptoms. Given the patient’s unresponsive state and the tumor’s rapid growth, chemotherapy was initiated under critical conditions. A combination of cyclophosphamide (1300 mg, day 1, every 4 weeks), vincristine (2 mg, day 1, every 4 weeks), and dacarbazine (1000 mg, day 1-2, every 4 weeks) was started in September 2022.Surprisingly, a CT scan revealed significant regressions of the retroperitoneal mass and lung metastases (**Figures 1B, E, H, K**) after two treatment cycles. According to the Response Evaluation Criteria in Solid Tumors version 1.1 (RECIST 1.1), a partial response (PR) was achieved. Additionally, a completely biochemical response with symptom remission was observed, allowing the cessation of symptomatic therapies (**Supplementary Figure 1**). NSE and catecholamine levels decreased concurrently (**Supplementary Figure 1**). In September 2024, CT scan revealed new pulmonary lesions and some lung lesion enlarged, suggesting disease progression. However, retroperitoneal lesions and the majority of lung lesions further reduced in size (**Figures 1C, F, I, L**). The progression-free survival (PFS) of CVD chemotherapy was 24 months. Given the high SSTR expression, a switch to somatostatin analogue (SSA) therapy is recommended. The patient remains stable without symptoms or treatment-related adverse effects.

## Discussion

In this case, we report a 40-year-old male patient with SDHB-mutated metastatic PGL. A rapid, deep and durable PR, and complete biochemical response were achieved after CVD chemotherapy.

The prevalence of the SDHB mutated PGLs among Chinese patients has been well-documented (7, 8). Compared to others, PGL with SDHB mutation typically exhibit an early onset, noradrenergic or dopaminergic biochemical phenotype, and shorter survival (9, 10). Additionally, precision medicine can be tailored based on the SDHB mutation status. Several studies have proved that CVD chemotherapy was the first-line treatment for PGL individuals with SDHB-mutation (11, 12). Our case reaffirms above points.

Missense mutations and truncating mutations are the most commonly reported types of SDHB mutations in PGLs (13). Compared to missense mutations, truncating mutations in SDHB are typically associated with a higher malignancy potential in PPGLs (14). In our study, the patient had a SDHB mutation characterized by a copy number loss, a mutation type that has not been widely reported in previous studies. While missense mutations and truncating mutations often lead to a complete loss of the biological function of key proteins, copy number loss typically results in a reduction in gene expression, causing partial functional loss. Whether this mutation type is associated with a better prognosis in patients remains to be confirmed through large-scale clinical studies.

Notably, SDHB mutation differs from the negative immunohistochemical expression of SDHB (15). Consequently, the lack of immunohistochemical expression of SDHB is often utilized as an alternative marker in assessing SDHx gene mutations (15–21). This approach is not only cost-effective but also aids in identifying false negative results from genetic testing. However, we believe that the negative immunohistochemical expression of SDHB cannot replace next-generation sequencing. Many studies have shown that different mutations in the SDHx gene often exhibit different clinical manifestations, which are of great significance for the prognosis and treatment of patients (22, 23). Therefore, we emphasize the significance of incorporating both genetic testing and immunohistochemical analysis in clinical practice for precise diagnosis and prognosis.

Due to the hyperglycemic effect of catecholamines, hypoglycemia directly induced by PGLs is exceedingly rare. We reviewed related profiles and concluded four mechanisms to identify the mechanisms underlying this hypoglycemia (**Supplementary Table S1**) (24–33). Firstly, tumor autoimmune hypoglycemia, often associated with myeloma and Hodgkin’s disease (34, 35). Secondly, liver, adrenal, or pituitary insufficiency also can contribute to hypoglycemia. Thirdly, a massive tumor burden may lead to rapid glucose consumption and subsequent hypoglycemia (29). Furthermore, the production of hypoglycemic substances by tumor, such as IGF-II, IGF-I and GH, can cause hypoglycemia (36). In our case, tumor- autoimmune hypoglycemia antibodies, liver function, adrenal function, and pituitary function were all normal, thus excluding the first two mechanisms. Given the patient’s high tumor burden and the improvement of hypoglycemia as the tumor shrank, the latter two mechanisms were assumed to be involved. Rapidly growing tumors consume glucose and release hypoglycemic substances, causing the rare complication of hypoglycemia. However, due to technical limitations and our current understanding, we unfortunately did not measure serum IGF-II levels during hypoglycemia. Therefore, we can only speculate that the causes of this hypoglycemia are likely multifactorial.

## Conclusion

This study presents a rare case of SDHB-mutated metastatic PGLs, demonstrating a rapid, deep and durable response to CVD chemotherapy. It underscored the critical role of SDHB mutations in influencing both prognosis and treatment selection for PGLs.

## Data Availability

The raw data supporting the conclusions of this article will be made available by the authors, without undue reservation.

## References

[1] Buffet A Burnichon N Favier J Gimenez-Roqueplo AP. An overview of 20 years of genetic studies in pheochromocytoma and paraganglioma. Best Pract Res Clin Endocrinol Metab. (2020) 34:101416. doi: 10.1016/j.beem.2020.101416 32295730

[2] Currás-Freixes M Piñeiro-Yañez E Montero-Conde C Apellániz-Ruiz M Calsina B Mancikova V. Pheoseq: A targeted next-generation sequencing assay for pheochromocytoma and paraganglioma diagnostics. J Mol diagnostics: JMD. (2017) 19:575–88. doi: 10.1016/j.jmoldx.2017.04.009 PMC5500830 28552549

[3] Ben Aim L Pigny P Castro-Vega LJ Buffet A Amar L Bertherat J. Targeted next-generation sequencing detects rare genetic events in pheochromocytoma and paraganglioma. J Med Genet. (2019) 56:513–20. doi: 10.1136/jmedgenet-2018-105714 30877234

[4] Gimenez-Roqueplo AP Favier J Rustin P Rieubland C Crespin M Nau V. Mutations in the sdhb gene are associated with extra-adrenal and/or Malignant phaeochromocytomas. Cancer Res. (2003) 63:5615–21. Available online at: https://aacrjournals.org/cancerres/article/63/17/5615/510352/Mutations-in-the-SDHB-Gene-Are-Associated-with#related-urls. 14500403

[5] Amar L Baudin E Burnichon N Peyrard S Silvera S Bertherat J. Succinate dehydrogenase B gene mutations predict survival in patients with Malignant pheochromocytomas or paragangliomas. J Clin Endocrinol Metab. (2007) 92:3822–8. doi: 10.1210/jc.2007-0709 17652212

[6] Cui Y Ma X Wang F Wang H Zhou T Chen S. Differences in clinical manifestations and tumor features between metastatic pheochromocytoma/paraganglioma patients with and without germline sdhb mutation. Endocrine practice: Off J Am Coll Endocrinol Am Assoc Clin Endocrinologists. (2021) 27:348–53. doi: 10.1016/j.eprac.2020.09.015 34024343

[7] Li C Li J Han C Wang T Zhang L Wang Z. Novel and recurrent genetic variants of vhl, sdhb, and ret genes in chinese pheochromocytoma and paraganglioma patients. Front Genet. (2023) 14:959989. doi: 10.3389/fgene.2023.959989 36936415 PMC10020357

[8] Ma X Li M Tong A Wang F Cui Y Zhang X. Genetic and clinical profiles of pheochromocytoma and paraganglioma: A single center study. Front Endocrinol. (2020) 11:574662. doi: 10.3389/fendo.2020.574662 PMC7761866 33362715

[9] Taïeb D Nölting S Perrier ND Fassnacht M Carrasquillo JA Grossman AB. Management of phaeochromocytoma and paraganglioma in patients with germline sdhb pathogenic variants: an international expert consensus statement. Nat Rev Endocrinol. (2024) 20:168–84. doi: 10.1038/s41574-023-00926-0 38097671

[10] Crona J Lamarca A Ghosal S Welin S Skogseid B Pacak K. Genotype-phenotype correlations in pheochromocytoma and paraganglioma: A systematic review and individual patient meta-analysis. Endocrine-related Cancer. (2019) 26:539–50. doi: 10.1530/erc-19-0024 PMC6717695 30893643

[11] Fishbein L Ben-Maimon S Keefe S Cengel K Pryma DA Loaiza-Bonilla A. Sdhb mutation carriers with Malignant pheochromocytoma respond better to cvd. Endocrine-related Cancer. (2017) 24:L51–l5. doi: 10.1530/erc-17-0086 28566531

[12] Jawed I Velarde M Därr R Wolf KI Adams K Venkatesan AM. Continued tumor reduction of metastatic pheochromocytoma/paraganglioma harboring succinate dehydrogenase subunit B mutations with cyclical chemotherapy. Cell Mol Neurobiol. (2018) 38:1099–106. doi: 10.1007/s10571-018-0579-4 PMC5976545 29623478

[13] Liu C Zhou D Yang K Xu N Peng J Zhu Z. Research progress on the pathogenesis of the sdhb mutation and related diseases. Biomedicine pharmacotherapy = Biomedecine pharmacotherapie. (2023) 167:115500. doi: 10.1016/j.biopha.2023.115500 37734265

[14] Bayley JP Bausch B Jansen JC Hensen EF van der Tuin K Corssmit EP. Sdhb variant type impacts phenotype and Malignancy in pheochromocytoma-paraganglioma. J Med Genet. (2023) 60:25–32. doi: 10.1136/jmedgenet-2020-107656 34750193

[15] Gill AJ. Succinate dehydrogenase (Sdh)-deficient neoplasia. Histopathology. (2018) 72:106–16. doi: 10.1111/his.13277 29239034

[16] Gill AJ Benn DE Chou A Clarkson A Muljono A Meyer-Rochow GY. Immunohistochemistry for sdhb triages genetic testing of sdhb, sdhc, and sdhd in paraganglioma-pheochromocytoma syndromes. Hum Pathol. (2010) 41:805–14. doi: 10.1016/j.humpath.2009.12.005 20236688

[17] Castelblanco E Santacana M Valls J de Cubas A Cascón A Robledo M. Usefulness of negative and weak-diffuse pattern of sdhb immunostaining in assessment of sdh mutations in paragangliomas and pheochromocytomas. Endocrine Pathol. (2013) 24:199–205. doi: 10.1007/s12022-013-9269-4 24096807

[18] Pai R Manipadam MT Singh P Ebenazer A Samuel P Rajaratnam S. Usefulness of succinate dehydrogenase B (Sdhb) immunohistochemistry in guiding mutational screening among patients with pheochromocytoma-paraganglioma syndromes. APMIS: Acta pathologica microbiologica immunologica Scandinavica. (2014) 122:1130–5. doi: 10.1111/apm.12269 24735130

[19] Giubellino A Lara K Martucci V Huynh T Agarwal P Pacak K. Urinary bladder paragangliomas: how immunohistochemistry can assist to identify patients with sdhb germline and somatic mutations. Am J Surg Pathol. (2015) 39:1488–92. doi: 10.1097/pas.0000000000000534 PMC4606469 26457353

[20] Oudijk L Gaal J de Krijger RR. The role of immunohistochemistry and molecular analysis of succinate dehydrogenase in the diagnosis of endocrine and non-endocrine tumors and related syndromes. Endocrine Pathol. (2019) 30:64–73. doi: 10.1007/s12022-018-9555-2 30421319

[21] Su T Yang Y Jiang L Xie J Zhong X Wu L. Sdhb immunohistochemistry for prognosis of pheochromocytoma and paraganglioma: A retrospective and prospective analysis. Front Endocrinol. (2023) 14:1121397. doi: 10.3389/fendo.2023.1121397 PMC10061060 37008946

[22] Kaplan AI Dwight T Luxford C Benn DE Clifton-Bligh RJ. Sdha related phaeochromocytoma and paraganglioma: review and clinical management. Endocrine-related Cancer. (2024). doi: 10.1530/erc-24-0111 PMC11466202 39133175

[23] Andrews KA Ascher DB Pires DEV Barnes DR Vialard L Casey RT. Tumour risks and genotype-phenotype correlations associated with germline variants in succinate dehydrogenase subunit genes sdhb, sdhc and sdhd. J Med Genet. (2018) 55:384–94. doi: 10.1136/jmedgenet-2017-105127 PMC5992372 29386252

[24] Mete O Asa SL Gill AJ Kimura N de Krijger RR Tischler A. Overview of the 2022 who classification of paragangliomas and pheochromocytomas. Endocrine Pathol. (2022) 33:90–114. doi: 10.1007/s12022-022-09704-6 35285002

[25] Hiramatsu K Takahashi K Kanemoto N Arimori S. A case of pheochromocytoma with transient hyperinsulinemia and reactive hypoglycemia. Japanese J Med. (1987) 26:88–90. doi: 10.2169/internalmedicine1962.26.88 3573413

[26] Fujino K Yamamoto S Matsumoto M Sunada M Ota T. Paraganglioma associated with hypoglycemia. Internal Med (Tokyo Japan). (1992) 31:1239–41. doi: 10.2169/internalmedicine.31.1239 1286235

[27] Uysal M Temiz S Gul N Yarman S Tanakol R Kapran Y. Hypoglycemia due to ectopic release of insulin from a paraganglioma. Hormone Res. (2007) 67:292–5. doi: 10.1159/000099291 17284922

[28] Frankton S Baithun S Husain E Davis K Grossman AB. Phaeochromocytoma crisis presenting with profound hypoglycaemia and subsequent hypertension. Hormones (Athens Greece). (2009) 8:65–70. doi: 10.14310/horm.2002.1224 19269923

[29] Habra MA Núñez R Chuang H Ayala-Ramirez M Rich T Kyle K. Fatal hypoglycemia in Malignant pheochromocytoma: direct glucose consumption as suggested by (18)F-2-fluoro-2-deoxy-D-glucose positron emission tomography/computed tomography imaging. Endocrine. (2010) 37:209–12. doi: 10.1007/s12020-009-9300-1 20963572

[30] Altincik A Ozen S Celik A Dokumcu Z Darcan S Abaci A. Pediatric bilateral pheochromocytoma and experience of laparoscopic cortical sparing adrenalectomy. J Pediatr Res. (2018) 5. doi: 10.4274/jpr.87486

[31] Martínez García M Trincado Aznar P López Alaminos ME González Fernández M Alvarado Rosas A Laclaustra Gimeno M. Persistent hypoglycemia due to an igf-ii-secreting Malignant pheochromocytoma: A case report and literature review. Clin Case Rep. (2020) 8:2433–5. doi: 10.1002/ccr3.3161 PMC7752311 33363755

[32] Abdulhadi B Anastasopoulou C Lekprasert P. Tumor-induced hypoglycemia: an unusual case report and review of literature. AACE Clin Case Rep. (2021) 7:80–3. doi: 10.1016/j.aace.2020.11.002 PMC7924146 33851027

[33] Alnahas Z Horani MH. Psun09 an unusual presentation of pheochromocytoma with persistent hypoglycemia, a case report. J Endocrine Soc. (2022) 6:A120. doi: 10.1210/jendso/bvac150.244

[34] Sorlini M Benini F Cravarezza P Romanelli G. Hypoglycemia, an atypical early sign of hepatocellular carcinoma. J Gastrointestinal Cancer. (2010) 41:209–11. doi: 10.1007/s12029-010-9137-0 20204540

[35] Lau CI Wang HC Hsu WC. Hypoglycemic encephalopathy as the initial presentation of hepatic tumor: A case report. Neurologist. (2010) 16:206–7. doi: 10.1097/NRL.0b013e3181a6ec56 20445433

[36] Bodnar TW Acevedo MJ Pietropaolo M. Management of non-islet-cell tumor hypoglycemia: A clinical review. J Clin Endocrinol Metab. (2014) 99:713–22. doi: 10.1210/jc.2013-3382 PMC5393479 24423303

