# Supplementary material for: Durable and deep response to CVD chemotherapy in SDHB-mutated metastatic paraganglioma: case report
Source: Front Endocrinol (Lausanne). 2024 Dec 18;15:1483516. doi: 10.3389/fendo.2024.1483516 (PMC11688215; doi:10.3389/fendo.2024.1483516)
Supplement: Supplementary file 1 [file DataSheet1.pdf]

Supplementary Material

Supplementary Tables

Table S1 cases of hypoglycemia induced by PGLs.

Case (issue year)	Age/Sex	Main complaint	primary tumour sites	metastasis	Presence of CA secretion	Plasma insulin during hypoglycaemia	Plasma C peptide during hypoglycaemia	IGF-I during hypoglycaemia	IGF-II during hypoglycaemia	IGF-II: IGF-I	Ref.
1987	57/ Male	Hypoglycaemia without hypertension	Adrenal	none	Increased normetanephrine and metanephrine secretion	Higher than normal	Higher than normal	N/D	N/D	N/D	(1)
1992	63/ Female	Hypoglycaemia without hypertension	Extra-adrenal	none	Increased normetanephrine secretion	Normal	Normal	N/D	N/D	N/D	(2)
2007	67/ Female	Hypertension and hypoglycaemia	Extra-adrenal	none	Normal	Higher than normal	Higher than normal	N/D	N/D	N/D	(3)
2009	47/ Male	Hypertension and hypoglycaemia	Adrenal	none	N/D	Higher than normal	Higher than normal	Normal	N/D	N/D	(4)
2010	30/ Male	Hypoglycaemia without hypertension	Adrenal	Liver, bones	Normal	Normal	Lower than normal	Lower than normal	Lower than normal	15.6	(5)
2018	15/ Female	Hypoglycaemia without hypertension	Adrenal	none	Normal	Higher than normal	Higher than normal	N/D	N/D	N/D	(6)
2020	69/ Female	Hypertension and hypoglycaemia	Adrenal	Bones, lungs, liver	Increased normetanephrine, metanephrine, 3-	Normal	Normal	Lower than normal	Normal	>45	(7)

					mexoxytyramine secretion						
2021	33/ Male	Hypoglycaemia without hypertension	Adrenal	Liver and lymphadenopathy	Increased normetanephrine secretion	Lower than normal	Lower than normal	Lower than normal	Normal	12:1	(8)
2022	27/ Female	Hypoglycaemia without hypertension	Adrenal	none	Increased normetanephrine and metanephrine secretion	Normal	Normal	N/D	N/D	N/D	(9)
Present case	40/ Male	Hypertension and hypoglycaemia	Extra-adrenal	Bones, lungs, liver	Increased norepinephrine, normetanephrine, 3-mexoxytyramine secretion	Normal	Normal	Normal	N/D	N/D	
CT, computed tomography; CA, catecholamine; N/D, not described; IGF-1, Insulin-like growth factor 1; IGF-2, Insulin-like growth factor 2											

1. Hiramatsu K, Takahashi K, Kanemoto N, Arimori S. A Case of Pheochromocytoma with Transient Hyperinsulinemia and Reactive Hypoglycemia. *Japanese journal of medicine* (1987) 26(1):88-90. Epub 1987/02/01. doi: 10.2169/internalmedicine1962.26.88.

2. Fujino K, Yamamoto S, Matsumoto M, Sunada M, Ota T. Paraganglioma Associated with Hypoglycemia. *Internal medicine (Tokyo, Japan)* (1992) 31(10):1239-41. Epub 1992/10/01. doi: 10.2169/internalmedicine.31.1239.

3. Uysal M, Temiz S, Gul N, Yarman S, Tanakol R, Kapran Y. Hypoglycemia Due to Ectopic Release of Insulin from a Paraganglioma. *Hormone research* (2007) 67(6):292-5. Epub 2007/02/08. doi: 10.1159/000099291.

4. Frankton S, Baithun S, Husain E, Davis K, Grossman AB. Phaeochromocytoma Crisis Presenting with Profound Hypoglycaemia and Subsequent Hypertension. *Hormones (Athens, Greece)* (2009) 8(1):65-70. Epub 2009/03/10. doi: 10.14310/horm.2002.1224.

5. Habra MA, Núñez R, Chuang H, Ayala-Ramirez M, Rich T, Kyle K, et al. Fatal Hypoglycemia in Malignant Pheochromocytoma: Direct Glucose Consumption as Suggested by (18)F-2-Fluoro-2-Deoxy-D-Glucose Positron Emission Tomography/Computed Tomography Imaging. *Endocrine* (2010) 37(1):209-12. Epub 2010/10/22. doi: 10.1007/s12020-009-9300-1.

6. Altincik A, Ozen S, Celik A, Dokumcu Z, Darcan S, Abaci A, et al. Pediatric Bilateral Pheochromocytoma and Experience of Laparoscopic Cortical Sparing Adrenalectomy. *The Journal of Pediatric Research* (2018) 5. doi: 10.4274/jpr.87486.

7. Martínez García M, Trincado Aznar P, López Alaminos ME, González Fernández M, Alvarado Rosas A, Laclaustra Gimeno M. Persistent Hypoglycemia Due to an Igf-Ii-Secreting Malignant Pheochromocytoma: A Case Report and Literature Review. *Clinical case reports* (2020) 8(12):2433-5. Epub 2020/12/29. doi: 10.1002/ccr3.3161.

8. Abdulhadi B, Anastasopoulou C, Lekprasert P. Tumor-Induced Hypoglycemia: An Unusual Case Report and Review of literature. *AACE clinical case reports* (2021) 7(1):80-3. Epub 2021/04/15. doi: 10.1016/j.aace.2020.11.002.

9. Alnahas Z, Horani MH. Psun09 an Unusual Presentation of Pheochromocytoma with Persistent Hypoglycemia, a Case Report. *Journal of the Endocrine Society* (2022) 6(Suppl 1):A120. doi: 10.1210/jendso/bvac150.244.

Supplementary figures

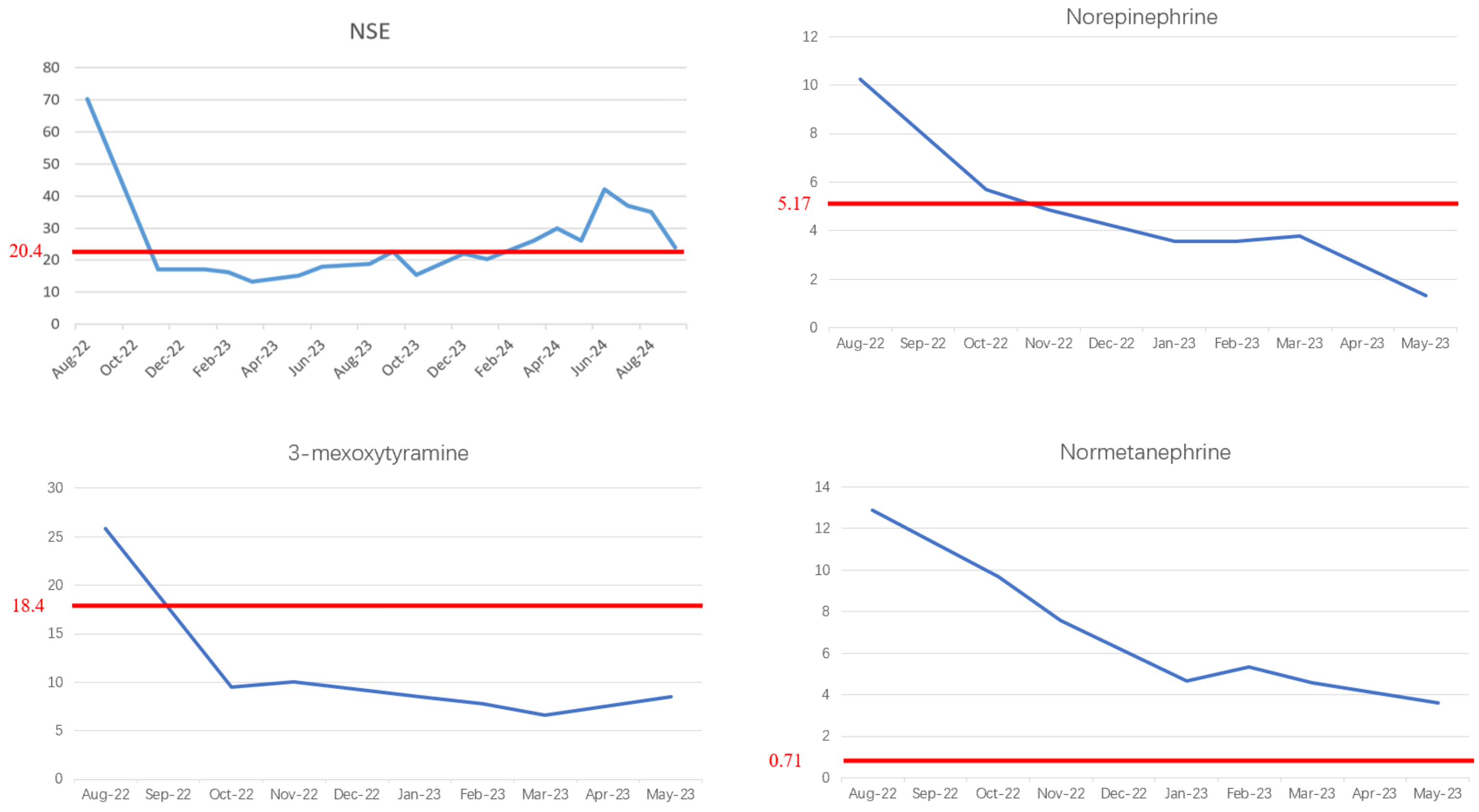

Supplementary figure 1 The trend in the patient's NSE, norepinephrine, 3-mexoxytyramine, normetanephrine levels. The red line indicates the upper limit of the normal value.

CARE case report guidelines

CARE Checklist of information to include when writing a case report

(cc) BY-NC-ND

✓

Topic	Item	Checklist item description	Reported on Line
Title	1	The diagnosis or intervention of primary focus followed by the words "case report"	Title
Key Words	2	2 to 5 key words that identify diagnoses or interventions in this case report, including "case report"	Key words
Abstract (no references)	3a	Introduction: What is unique about this case and what does it add to the scientific literature?	Abstract
	3b	Main symptoms and/or important clinical findings	Abstract
	3c	the main diagnoses, therapeutic interventions, and outcomes	Abstract
	3d	Conclusion—What is the main "take-away" lesson(s) from this case?	Abstract
Introduction	4	One or two paragraphs summarizing why this case is unique (**may include references**)	Introduction
Patient Information	5a	De-identified patient specific information	Case presentation
	5b	Primary concerns and symptoms of the patient	Case presentation
	5c	Medical, family, and psycho-social history including relevant genetic information	Case presentation
	5d	Relevant past interventions with outcomes	Case presentation
Clinical Findings	6	Describe significant physical examination (PE) and important clinical findings	Case presentation
Timeline	7	Historical and current information from this episode of care organized as a timeline	Figure 1
Diagnostic Assessment	8 8a	Diagnostic testing (such as PE, laboratory testing, imaging, surveys).	Case presentation
	8b	Diagnostic challenges (such as access to testing, financial, or cultural)	Case presentation
	8c	Diagnosis (including other diagnoses considered)	Case presentation
	8d	Prognosis (such as staging in oncology) where applicable	Case presentation
Therapeutic Intervention	9a	Types of therapeutic intervention (such as pharmacologic, surgical, preventive, self-care)	Case presentation
	9b	Administration of therapeutic intervention (such as dosage, strength, duration)	Case presentation
	9c	Changes in therapeutic intervention (with rationale)	Case presentation
Follow-up and Outcomes	10a	Clinician and patient-assessed outcomes (if available)	Case presentation
	10b	Important follow-up diagnostic and other test results	Case presentation
	10c	Intervention adherence and tolerability (How was this assessed?)	Case presentation
	10d	Adverse and unanticipated events	Case presentation
Discussion	11a	A scientific discussion of the strengths AND limitations associated with this case report	Discussion
	11b	Discussion of the relevant medical literature **with references**	Discussion
	11c	The scientific rationale for any conclusions (including assessment of possible causes)	Conclusion
	11d	The primary "take-away" lessons of this case report (without references) in a one paragraph conclusion	Conclusion
Patient Perspective	12	The patient should share their perspective in one to two paragraphs on the treatment(s) they received	no
Informed Consent	13	Did the patient give informed consent? Please provide if requested	Yes ☑ No ☐
